# The telomere tumult: meaning and metrics in population studies

**DOI:** 10.1016/s2666-7568(22)00094-0

**Published:** 2022-05-04

**Authors:** Abraham Aviv

**Affiliations:** The Center of Human Development and Aging, New Jersey Medical School, Rutgers University, Newark, NJ 07103, USA

In *The Lancet Healthy Longevity*, Vasiliki Bountziouka and collegaues^1^ have authored the latest in a series of communications that leveraged the UK Biobank to transform population-based telomere research. Their paper focuses on associations, in about 400 000 UK Biobank participants, between potentially modifiable traits and leukocyte telomere length (LTL). It shows that behaviours explain less than 0·2% of the inter-individual LTL variation and exert no effect on LTL association with risks of 22 diseases causally linked to it. These findings reinforce a previous meta-analysis by Pepper and colleagues,^2^ who concluded, based on data from about 400 000 people, that exposures and stress, principally psychological stress, are only weakly associated with LTL. Pepper and colleagues further indicated that such associations might be driven by publication bias and underpowered sample sizes.

Jointly, these large studies question the present focus of population studies, reflected in the US National Institutes of Health announcements on Telomeres as sentinels of environmental exposures, psychosocial stress, and disease susceptibility, and on Telomeres in wellness and disease: a biobehavioral approach. The premise driving these announcements is that exposures adversely affecting health also shorten LTL, thereby increasing LTL-related disease risk. However, Bountziouka and colleagues show that common exposures and behaviours are unlikely to exert a consequential influence on LTL during adulthood.

Evolution works through gene–environment interaction. Accordingly, contemporary LTL reflects past trade-offs across opposing forces. These included different exposures that resulted in telomere lengthening or shortening to an optimal length for a given geography and evolutionary period.^3^ However, the characteristics of these forces were shaped by elements that largely differ from those affecting fitness in present upper-middle-income populations.

The nominal effect of exposures and behaviours on LTL is, therefore, hardly surprising. The vast person-to-person LTL variation at any age (SD about 700 bp, range about 3000 bp) is determined before adulthood, principally at birth.^4,5^ This concept draws on LTL measurements by Southern blotting showing that, first, the inter-individual LTL variation among newborns is like that among their parents^5^ and, second, the inter-individual differences in LTL shortening after the first decade of life do not offset LTL ranking across individuals, which is established before adulthood.^6,7^ Thus, LTL tracks with age, meaning that individuals entering adulthood with short (or long) LTL maintain their short (or long) LTL for their remaining lives. Collectively, findings that LTL ranking precedes disease onset by decades^4,5^ and genetic evidence, including Mendelian randomisation analysis,^8^ support a causal role of LTL in some human diseases. Bountziouka and colleagues thus propose that population studies pivot away from their focus on behaviours and refocus instead on mechanisms explaining LTL causality in these diseases.

This proposition would not be possible were it not for the prescience and perseverance of the UK Biobank investigators, who used a high-throughput quantitative PCR (qPCR) method to generate a massive LTL database that offsets the method’s imprecision. By contrast, many investigators used the qPCR method indiscriminately in small studies, including longitudinal investigations optimal for testing causal effects of exposures and behaviours on LTL. Such studies demand precise telomere length measurements because the yearly LTL change among adults is two orders of magnitudes smaller than the absolute LTL, whose measurement error is introduced twice, at baseline and follow-up.^9^ Yet, based on qPCR data, some of these longitudinal studies have claimed that healthy behaviours slow age-related LTL shortening and might even lengthen LTL.^10^ Predictably, for-profit outfits have stepped in to offer non-expert consumers serial LTL measurements along with advice for maintaining telomere health.

Nonetheless, the paper by Bountziouka and colleagues reveals that sample size cannot make up for another major limitation of the qPCR method—ie, its telomere to single-gene ratio metric. This ratio simply indicates that LTL is shorter or longer in group A versus group B. To provide a quantitative context for their findings, the authors transformed their telomere to single-gene ratio into equivalent years of age-related change in LTL. However, the noise of transforming the ratio into this amorphous unit might amplify the qPCR imprecision rather than set a meaningful standard that can be used in clinical settings.

To conclude, behaviours and exposures, including psychological stress, exert inconsequential effects on LTL. The UK Biobank is a model for the proper application of the high-throughput qPCR method. By contrast, the wholesale use of the method for smaller studies and longitudinal ones has undermined telomere epidemiology. The method must be reserved for large studies, as major efforts are undertaken to develop precise, high-throughput techniques that generate data in absolute telomere length units. For now, however, investigators embarking on small studies, particularly longitudinal studies, might consider precise gel-based and flow fluorescent in situ hybridisation techniques for LTL measurements. Notwithstanding their labour-intensive nature, these techniques have generated most of our knowledge about the role of telomeres in human health, from the molecular to the population levels.
